# Trends in Fouling Resistant Membranes Containing Metals or Metallic Nanoparticles for the Separation of Oil-in-Water Emulsions

**DOI:** 10.1021/acsomega.4c10000

**Published:** 2025-02-21

**Authors:** Felipe P. da Silva, Cristiano P. Borges, Fabiana V. da Fonseca

**Affiliations:** † Chemistry School, Federal University of Rio de Janeiro, Av. Athos da Silveira Ramos 149, Rio de Janeiro, Rio de Janeiro 21941-909, Brazil; ‡ Alberto Luiz Coimbra Institute for Graduate Studies and Research in Engineering (COPPE), Federal University of Rio de Janeiro, Av. Horácio Macedo 2030, Rio de Janeiro, Rio de Janeiro 21941-972, Brazil

## Abstract

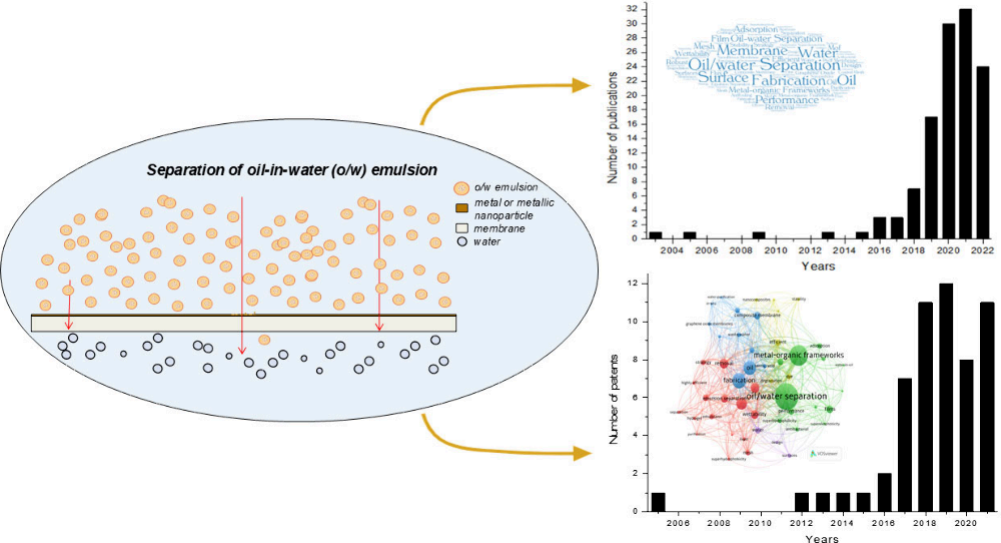

Oil production entails the generation of large volumes of complex effluents that contain emulsified oil in water. Difficult to manage, the use of membrane separation processes (MSP) is an interesting option for the treatment of oily effluents, separating oil-in-water (o/w) emulsions with high efficiency. Modifying the surface of membranes with metals minimizes fouling, which is the main drawback of MSP. Through a survey of scientific papers and patent applications, this review demonstrates the technological development of membranes containing metals or metallic nanoparticles (MNP) for separating o/w emulsions. In total, 314 articles and 339 patent filings were retrieved by July 2023. The beginning of growth in the number of documents retrieved was more significant in 2018 for articles and in 2017 for patents. Asia was the largest highlight, with emphasis on China, which had 76.97% of the production of articles and 98.31% of patent applications on the continent, justified by investment in Research and Development (R&D). Metals, such as Fe, Zn, Cu, and Ti have been used to modify membranes and substrates. Polyvinylidene fluoride (PVDF), polyacrylonitrile (PAN), and polypropylene (PP) membranes and carbon-based primary substrates were used for their preparation. Advancements in membranes containing metals or MNP for the separation of o/w emulsions can have a positive impact on Sustainable Development Goals (SDG). This developing field anticipates increased involvement in nanotechnology, the development of systems that enable both the separation and oxidation of organic compounds, and a variety of methods or combinations of approaches to synthesize increasingly efficient membranes.

## 1 Introduction

Oil is a primary source of energy and revenue worldwide.^1^ In the field, its occurrence is associated with gas and water under environmental conditions in which oil-in-water (o/w) emulsions are favored. Water injection in oil reservoirs is commonly used to adjust pressure and facilitate oil recovery. To enhance efficiency, chemical additives such as surfactants, demulsifiers, biocides, antifoaming agents, and antiscaling agents are often used.^2^ Produced water (PW), a byproduct of oil production, is formed by combining the injected water with the formation water present in the reservoir. Approximately 1200 chemical components have been detected in PW samples.^3^

The management of large volumes of this complex effluent is a major challenge for industry.^4^ In general, the main issue associated with PW is its oil and grease (O&G) content. For disposal, this parameter has a limit that varies according to country or group of countries. China has the strictest limit of 10 mg/L because of the country’s ambition to overcome pollution.^5,6^ Argentina, Venezuela, and the signatory countries of the Helsink Convention have restrictive limits. However, instead of establishing an average limit of 15 mg/L, they accepted an alternative monthly average of 40 mg/L as a criterion if the country was unable to use technologies that treat effluents at a value equal to or less than 15 mg/L. In Brazil, CONAMA Resolution 393/2007^7^ set a maximum average concentration of 29 mg/L, with a maximum value of 42 mg/L for discharge on offshore platforms, and CONAMA 430/2011^8^ set a maximum value of 20 mg/L for mineral oils in cases close to the coast.

The International Association of Oil and Gas Producers (IOGP) reported that the average O&G of PW discharges was 14.4 mg/L in 2021, compared to 16.4 mg/L in 2020 and 17.5 mg/L in 2019.^2^ In offshore operations, the O&G in PW in 2021 was 17.1 mg/L, whereas onshore it was 5.7 mg/L.^2^ However, oily wastewater poses severe risks. It is mutagenic and carcinogenic to humans, inhibits plant growth, elevates biochemical oxygen demand (BOD) and chemical oxygen demand (COD), and forms a sunlight-impermeable layer that disrupts aquatic ecosystems when discarded without treatment.^9^

As legislation on disposal becomes increasingly restrictive, and oil extraction occurs in water-stressed regions, treating and reusing oily wastewater offers significant environmental and economic benefits.^10−12^ Membrane separation processes (MSP) stand out among the various possible methods. These are based on the use of a selective physical barrier (membrane), which allows the passage of water and retention of undesirable compounds under the action of a driving force.

MSP offer several advantages, including simplicity of operation, low energy consumption, high productivity, scalability, and enhanced removal capacity.^13−16^ However, the deposition and accumulation of rejected materials, such as organic or inorganic compounds and microorganisms, on the surface and pores of the membrane severely reduces treatment efficiency and can lead to membrane loss.^17−19^ This problem is called fouling and depends on several factors, such as the characteristics of the feedwater, membrane properties (material, porosity, hydrophobicity, etc.), operating conditions, and concentration polarization.^17^ Membrane fouling is less intense in ceramic membranes than in their polymeric counterparts, owing to their hydrophilic surfaces.^20^ Among the strategies and techniques for fouling mitigation, the pretreatment of feedwater, optimization of operating conditions, membrane monitoring and cleaning, surface modification, and new membrane materials can be highlighted.^13−17^

Emerging techniques, such as surface modification and functionalization, aim to improve the antifouling properties of membranes, particularly ceramics.^13^ Polymeric nanocomposite membranes are modifications of conventional polymeric membranes with nanomaterials dispersed within their polymeric networks.^17^ Among the most commonly used materials for membrane modification are metals, such as iron (Fe), copper (Cu), titanium (Ti), cobalt (Co), silver (Ag), zinc (Zn), zirconium (Zr), and aluminum (Al).^21−32^ In another approach, a metal felt or mesh is used as a support for polymer composite hydrophilic ultrafiltration (UF) membranes to separate o/w emulsions.^33,34^

Many companies have invested in Research and Development (R&D) to develop new membranes for oily water treatment. In this sense, searching for technical information in databases and specialized literature is crucial for guiding and saving time and expenses.^35^ This indicates that the most viable scenarios can favor a solid decision about offering new business and growth opportunities.^36^ Thus, mapping the development of specific technologies and emerging markets facilitates this analysis. The general objective of technological prospecting is reinforced using bibliometric analysis, a method that quantitatively and statistically analyzes documents in a given field, and has been applied in engineering and environmental science research on topics such as water remediation, nanomaterials, and climate change.^37^

Various approaches for membranes containing metals or metallic nanoparticles (MNP) for o/w emulsion separation have been reported in the literature. Wang et al. (2018) reviewed the research progress on the fabrication of porous metal filter membranes.^38^ Di et al. (2021) provided an overview of cutting-edge research on fabricating and applying porous membranes, including those based on zeolite, metal–organic frameworks (MOF), porous organic materials (POM), and mesoporous materials.^39^ Chen et al. (2021) focused on modifying ceramic membranes, while Huang et al. (2021) explored recent advances in polymeric membrane surface coatings. Their research included plant polyphenols and transition metal ions to enhance the antifouling performance of o/w emulsion separation.^40^

To the best of our knowledge, mapping the technology of membranes containing metals or MNP for o/w emulsion separation is lacking in literature. Therefore, an analysis of scientific articles and patents retrieved from the Web of Science (WoS) and Derwent Innovation Index (DII) was used to better understand the development of the field. The retrieved documents were used to categorize the membranes according to the metal and base substrate used for their preparation. We demonstrate how advances in membranes containing metals or MNP for o/w emulsion separation can have a positive impact on Sustainable Development Goals (SDG) and on current and future developments.

## 2 Analysis of Articles and Patent Applications

**Figure 1:**
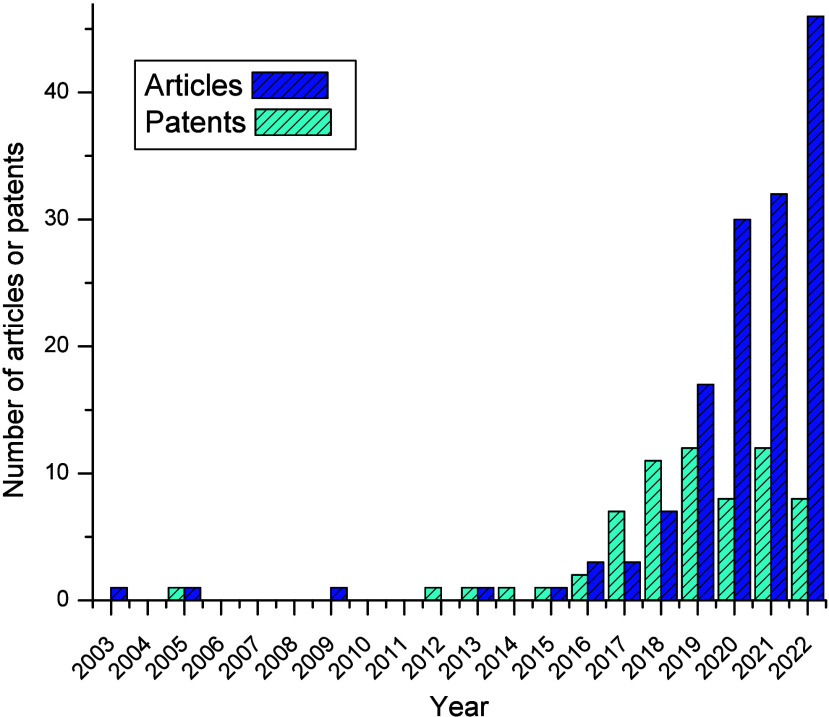
Time evolution of the published articles and patent applications on membranes containing metals or metallic nanoparticles to separate o/w emulsions in the WoS and DII databases until July 2023.

An initial search retrieved 314 articles and 339 patents. Of these, only 157 articles and 65 patent applications have focused on membranes containing metals or MNP for separating o/w emulsions. The first article was published in 2003 by Qiu and Zhang (2003) on the preparation of UF membranes composed of poly(vinyl alcohol) (PVA) coated on a metallic mesh.^41^ The earliest patent, Chinese CN1721030-A, filed in 2005, detailed the preparation of a superhydrophobic/superoleophilic o/w separation network. This network features meshes coated with a thin perfluoroalkyl siloxane copolymer membrane.^42^ Fourteen articles from 2023 were excluded due to incomplete data. Figure 1 shows the temporal evolution of the selected publications and number of filed patents.

As shown in Figure 1, research and patent applications for membranes containing metals or MNP exhibit a growing trend, reflecting strong interest in this topic. The increase in the number of publications became more pronounced in 2018, reaching a peak in 2022, with 46 publications (29.30% of the total). Similarly, patent applications saw a significant rise beginning in 2017 and peaking in 2019 with 12 patents (18.46% of the total). It is important to note that data from 2020 to 2022 may still evolve owing to the 18-month confidentiality period for patents, counted from the filing date, and delays in indexing documents in the database. The rapid growth in recent years suggests that the field is in a dynamic stage of development. The lower number of patent filings compared to published articles may indicate that research on membranes containing metals or MNP for o/w emulsion separation is incipient.

Academic-scientific development is associated with industrial interest in MSP, which are gaining ground because of their simplicity of operation, low energy consumption, high productivity, ease of scaling, and increased removal capacity.^13,15,16^ In addition, the world’s water is facing severe scarcity and pollution problems, particularly in India, Iran, Pakistan, and Turkmenistan, among others, which has increased interest in advanced water purification processes.^43,44^

**Figure 2:**
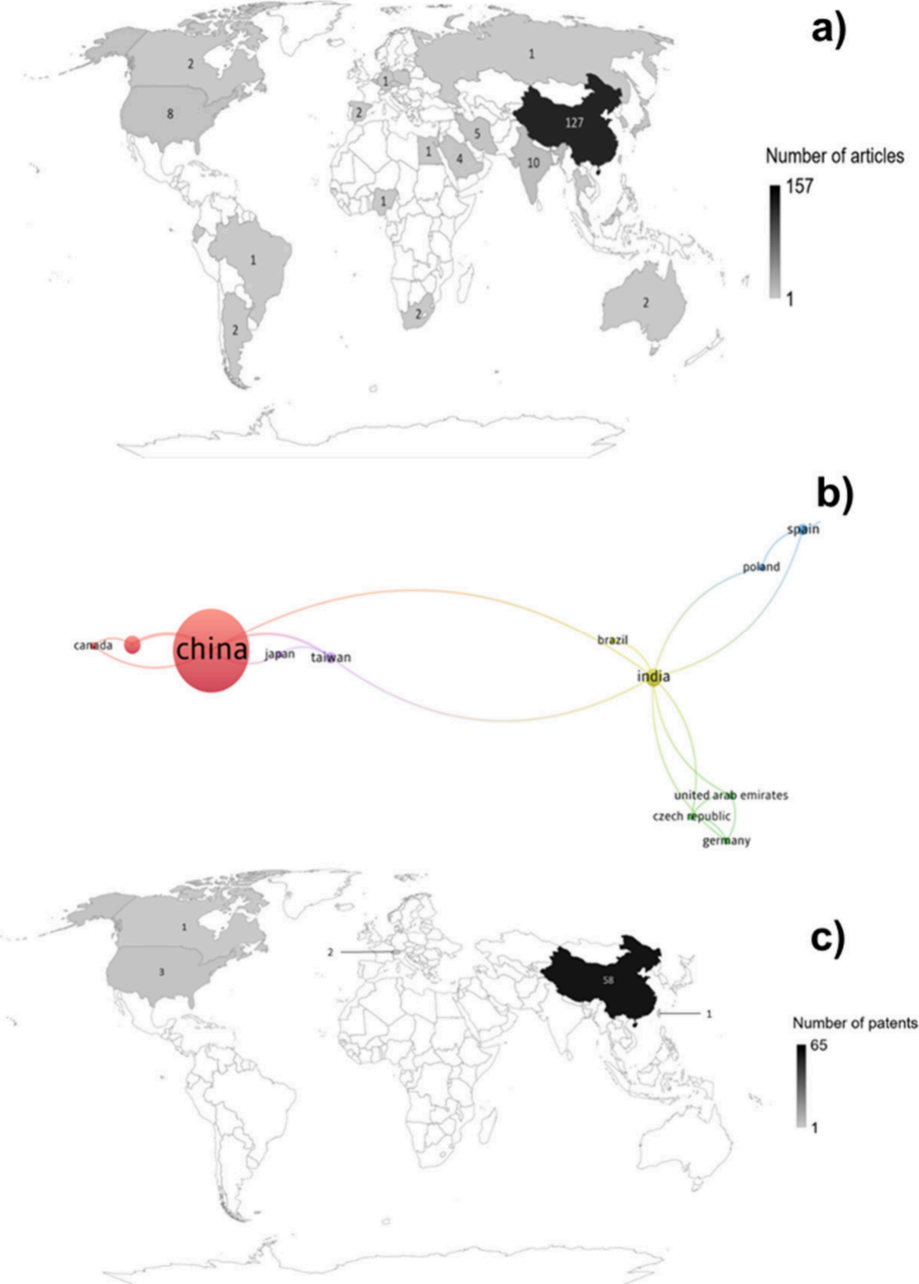
a) Geographical distribution of publications, b) cooperation of countries and regions on the research, and c) geographical distribution of patents filed on membranes containing metals or metallic nanoparticles to separate o/w emulsions retrieved from the WoS and DII databases until July 2023.

The analysis of geographic distribution provides a general idea of article production, as well as of the most significant shares of countries and regions interested in technology protection. Figure 2 shows the geographical distribution of publications, cooperation of countries and regions in the research, and distribution of patent applications.

As shown in Figure 2a, Asia has the largest share of publications, followed by North America and Europe. Within Asia, 76.97% of the publications originated from China, reflecting the country’s unprecedented industrialization, which has led to increased water use and a higher generation of liquid effluents. China’s leadership in this field is unsurprising because addressing pollution is one of the three main drivers of its development.^5^ Additionally, water scarcity, large-scale discharges, and increasingly stringent wastewater discharge standards^45^ have spurred substantial investments in membrane technology within the country. With the advent of nanotechnology, nanocomposite membranes have played a central role in o/w emulsion separation research.^22,24,46−49^

Along with China, India and the United States of America (USA) share 6.37% and 5.10% of publications, respectively, which is not surprising given the high level of water stress in both countries.^50^ Another justification for the superior production of these countries lies in investments in R&D and technology development, where the USA and China, for example, invest approximately 2.6% and 2.1% of their gross domestic product, respectively, for this purpose^51^ and perform several partnerships in research.^52^

To analyze international cooperation, data were collected from countries with at least one published article. Of the 157 selected articles, 50 (31.85%) indicated cooperation among different countries. In Figure 2b, each circle represents one country, indicating that international cooperation is broad. The larger the circles and thicker the lines, the more articles and links there are between the countries. Five clusters were formed: the red cluster was composed of China, the USA, and Canada; and the green cluster was composed of Germany, the Czech Republic, and the United Arab Emirates, who had one article in cooperation with India. The blue cluster includes Spain, Iran, and Poland; the yellow cluster includes Brazil and India, and the purple cluster includes Japan and Taiwan. Egypt, Saudi Arabia, and South Korea collaborate on one article but have no connection to others outside this group and are therefore excluded from viewing. China and India have many links to other countries.

As shown in Figure 2c, Asia has the largest share of patent filings (90.77%), followed by North America (6.15%), and Europe (3.08%). Notably, 98.31% of the priority patent applications in Asia were from China. This behavior is similar to the publications of articles previously shown, and is probably due to the investments of the Chinese government in the environmental area due to the resultant problems of industrialization.^5,51^ In addition to the applications filed in China, 2 patents were filed in Europe, and 3 of the 4 in North America were from the USA. Notably, research, development, and technology investment are substantial in North America.^51,52^

A total of 222 institutions collaborated with the research in this area. Table 1 classifies the 12 institutions with the most publications, accounting for 43.31% of the total number of publications. It is important to note that WoS performs the analysis considering all of the authors’ institutions, not just the first author.

**Table 1:** Most Productive Institutions in Publications Related to Membranes Containing Metals or Metallic Nanoparticles to Separate o/w Emulsions

	Institution	Country	Number of publications
1	Southwest Petroleum University	China	15
2	Chinese Academy of Sciences	China	7
3	Zhejiang University of Technology	China	7
4	China University of Petroleum	China	5
5	Jiangsu University	China	5
6	Soochow University China	China	5
7	Central South University	China	4
8	Collaborative Innovation Center of membrane separation and water treatment	China	4
9	Dalian University of Technology	China	4
10	National University of Singapore	Singapore	4
11	Razi University	Iran	4
12	Zhejiang University	China	4

Data in Table 1 demonstrate China’s interest in the area, where 10 of the 12 institutions on the list are from the country. An analysis of patent depositors revealed that 62 institutions created deposits in the area, with no significant innovations. This evidence indicates that there is plenty of room for research and patent development. The top 16 studies are presented in Table 2. It is important to emphasize that the DII database performs the count considering not only the first author’s institution.

**Table 2:** Principal Patent Applicants Related to Membranes Containing Metals or Metallic Nanoparticles for Separating o/w Emulsions

	Depositors	Type	Country	Number of patent applications
1	China Petroleum & Chemical Corporation	Company	China	4
2	Sinopec Beijing Research Institute of Chemical Industry Co., Ltd.	Institute or research center	China	4
3	Tianjin Polytechnic University	University	China	4
4	Jiangsu University	University	China	3
5	Jilin University	University	China	3
6	Central South University	University	China	2
7	China University Petroleum (East China)	University	China	2
8	Consejo Nacional Investigaciones Científicas y Técnicas	Institute or research center	Argentina	2
9	Guangdong University of Technology	University	China	2
10	Harbin Institute of Technology	University	China	2
11	Nanjing University	University	China	2
12	Ningbo Institute of Materials Technology and Engineering, Chinese Academy of Sciences	Institute or research center	China	2
13	University of Buenos Aires	University	Argentina	2
14	University of Electronic Science and Technology of China	University	China	2
15	Zhejiang Normal University	University	China	2
16	YPF Tecnología S.A.	Company	Argentina	2

As shown in Table 2, the number of universities is much higher than that of institutes, research centers, and companies. Among the listed depositors, only three were non-Chinese. They refer to deposits made in partnership with Argentine institutions, one of which is controlled by YPF,^53−55^ which is the country’s leading hydrocarbon exploration and production company.^56^

No depositors stand out from the rest in terms of deposit volume. The leading company is the China Petroleum & Chemical Corporation (SINOPEC), which, in partnership with the Sinopec Beijing Research Institute of Chemical Industry, had four^4^ deposits granted.^57−60^ It is one of the largest integrated energy and chemical companies in China, the number one refining company in the country, and the largest supplier of refined oil.^61^

Tianjin Polytechnic University, present-day Tiangong University, is a Chinese university with a key national laboratory for membranes and MSP.^62^ It was the top depositor university, with four patents.^29,63−65^ These facts corroborate the interest in the subject on the part of the country, primarily because of the water stress China is experiencing.^50^

**Figure 3:**
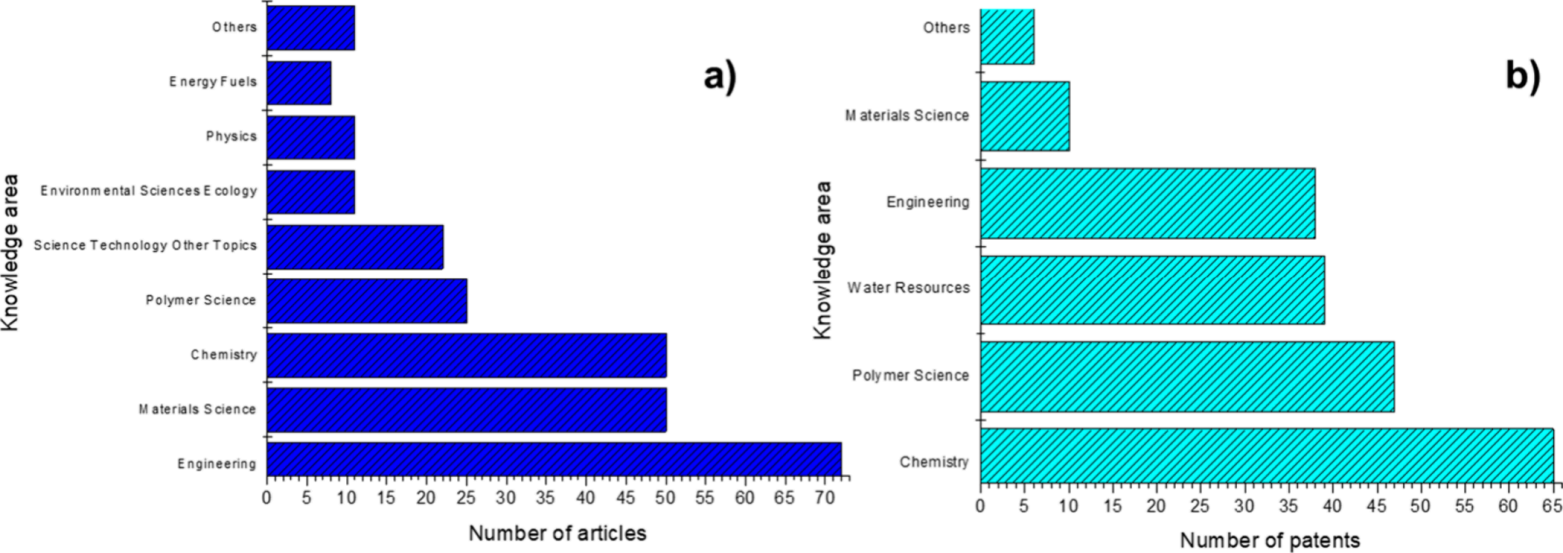
Knowledge areas involved in a) publications and b) patent applications on membranes containing metals or metallic nanoparticles to separate o/w emulsions found in the WoS and DII databases until July 2023.

Each article and patent on membranes containing metals or MNP for separating o/w emulsions was assigned to one or more knowledge areas. Figure 3 shows the distribution of the main areas according to the databases.

As shown in Figure 3a, interest in membranes containing metals or MNP for this application is interdisciplinary. The areas of engineering (45.86%), Materials Science (31.85%), and chemistry (31.85%) are prominent. This is due to the need to invest in more effective wastewater treatment solutions,^66,67^ which, concerning the use of membrane-based technologies, may involve the development of materials with improved hydrophilicity and wettability, among other properties.^22,30,68−72^ A total of 64 journals were responsible for publishing the selected documents, indicating a consistent distribution and broad interest in metal-containing membranes for this application.

As shown in Figure 3b, chemistry gained prominence (100.0%), including Polymer Science (72.3%) and Water Resources (60.0%). Given the inherent advantages of using MSP for water and effluent treatments, this is not surprising.^13−16^ Obtaining and modifying materials that can separate o/w emulsions is extremely important, particularly if certain advantages are associated.^22,30,68−72^

One of the most widely applied bibliometric indicators for assessing the quality of a given document is citation analysis. Table 3 presents the ten most-cited articles sorted by the total number of citations. Table 4 presents the five most-cited patents. The tables were sorted according to the total citations (TC) number.

**Table 3:** Most-Cited Publications on Membranes Containing Metals or Metallic Nanoparticles to Separate o/w Emulsions

	Title	Journal	TC	Average per Year	Reference
1	“*Nature-inspired chemistry toward hierarchical superhydrophobic, antibacterial and biocompatible nanofibrous membranes for effective UV-shielding, self-cleaning and oil-water separation*”	*Journal of Hazardous Materials*	195	48.75	(73)
2	“*Multiphase surface growth of hydrophobic ZIF-8 on melamine sponge for excellent oil/water separation and effective catalysis in a Knoevenagel reaction*”	*Journal of Materials Chemistry A*	164	27.33	(74)
3	“*Facile preparation of loess-coated membranes for multifunctional surfactant-stabilized oil-in-water emulsion separation*”	*Green Chemistry*	132	26.40	(75)
4	“*Nanofibrous metal-organic framework composite membrane for selective efficient oil/water emulsion separation*”	*Journal of Membrane Science*	111	15.86	(76)
5	“*Photo-Fenton self-cleaning PVDF/NH2-MIL-88B(Fe) membranes towards highly-efficient oil/water emulsion separation*”	*Journal of Membrane Science*	121	30.25	(77)
6	“*UiO-66-Coated Mesh Membrane with Underwater Superoleophobicity for High-Efficiency Oil-Water Separation*”	*ACS Applied Materials& Interfaces*	102	17.00	(78)
7	“*Self-assembled MOF membranes with underwater superoleophobicity for oil/water separation*”	*Journal of Membrane Science*	108	18.00	(21)
8	“*Influence of Surface Properties of Filtration-Layer Metal Oxide on Ceramic Membrane Fouling during Ultrafiltration of Oil/Water Emulsion*”	*Environmental Science& Technology*	98	12.25	(79)
9	“*An Ultrahydrophobic Fluorous Metal-Organic Framework Derived Recyclable Composite as a Promising Platform to Tackle Marine Oil Spills*”	*Chemistry-A European Journal*	78	11.14	(80)
10	“*TMU-5 metal-organic frameworks (MOFs) as a novel nanofiller for flux increment and fouling mitigation in PES ultrafiltration membrane*”	*Separation and Purification Technology*	89	14.83	(81)
	*High-efficiency separation performance of oil–water emulsions of polyacrylonitrile nanofibrous membrane decorated with metal–organic frameworks*	*Applied Surface Science*	85	17.00	(82)

**Table 4:** Most-Cited Patents on Membranes Containing Metals or Metallic Nanoparticles for Separating o/w Emulsions

	Title	TC	Average per Year	Patent Number	Reference
1	“*Super-hydrophobic/super-oleophilic oil-water separating net*”	43	2.39	CN1721030-A	(42)
2	“*Membranes the layer of metal organic framework particles*”	34	11.33	WO2019186134-A1	(83)
3	“*Separation membrane with superhydrophilic/underwater superoleophobicity, its preparation method and application*”	23	2.56	CN103100239-A	(84)
4	“*Underwater super-oleophobic oil-water separation mesh membrane and preparation method therefrom*”	21	2.33	CN102716676-A	(85)
5	“*A kind of magnetic response high- efficiency oil-water separation fiber membrane and preparation method therefrom*”	18	2.25	CN104436760-A	(86)

The article, “*Nature-inspired chemistry toward hierarchical superhydrophobic, antibacterial and biocompatible nanofibrous membranes for effective UV-shielding, self-cleaning and oil–water separation*” had both the highest number of citations and average per year. Ma et al. (2020) reported the fabrication of nanofibrous membranes that are eco-friendly, superhydrophobic, and inexpensive, with self-cleaning properties and UV protection.^73^

Highlights can also be given to the articles “*Facile preparation of loess-coated membranes for multifunctional surfactant-stabilized oil-in-water emulsion separation*”, “*Influence of Surface Properties of Filtration-Layer Metal Oxide on ceramic membrane fouling during ultrafiltration of Oil/Water Emulsion*”, and “*An ultrahydrophobic Fluorous Metal–Organic Framework Derived Recyclable Composite as a Promising Platform to Tackle Marine Oil Spills*”.^75,79,80^ Although these articles were not published in journals with the highest number of contributions to the field, they appeared in high-impact-factor periodicals. This likely enhances their visibility and increases their potential for a higher number of citations.^87^

The most-cited patent was the oldest among those who recovered for evaluation. The assignee responsible for this is Nanjing University, one of the top 16 depositors in the area (Table 4). Considering the average per year, patent number WO2019186134-A1 stands out.^83^ G2O Water Technologies Ltd. filed a patent, a UK-based graphene application, and an innovation company whose patented technology aims to reduce the cost of water treatment and filtration.^88^

The Chinese patent number CN103100239-A, the third most-cited, was filed by the Suzhou Institute of Nano-Tech and Nano-Bionics, an institute responsible for developing new materials and processes that can be transferred to industry.^84,89^ This patent focuses on preparing and employing a membrane separation network containing nanowires composed of cupric hydroxide or cupric oxide, which is another example of the direct application of nanotechnology to obtain membranes for the separation of o/w emulsions.

**Figure 4:**
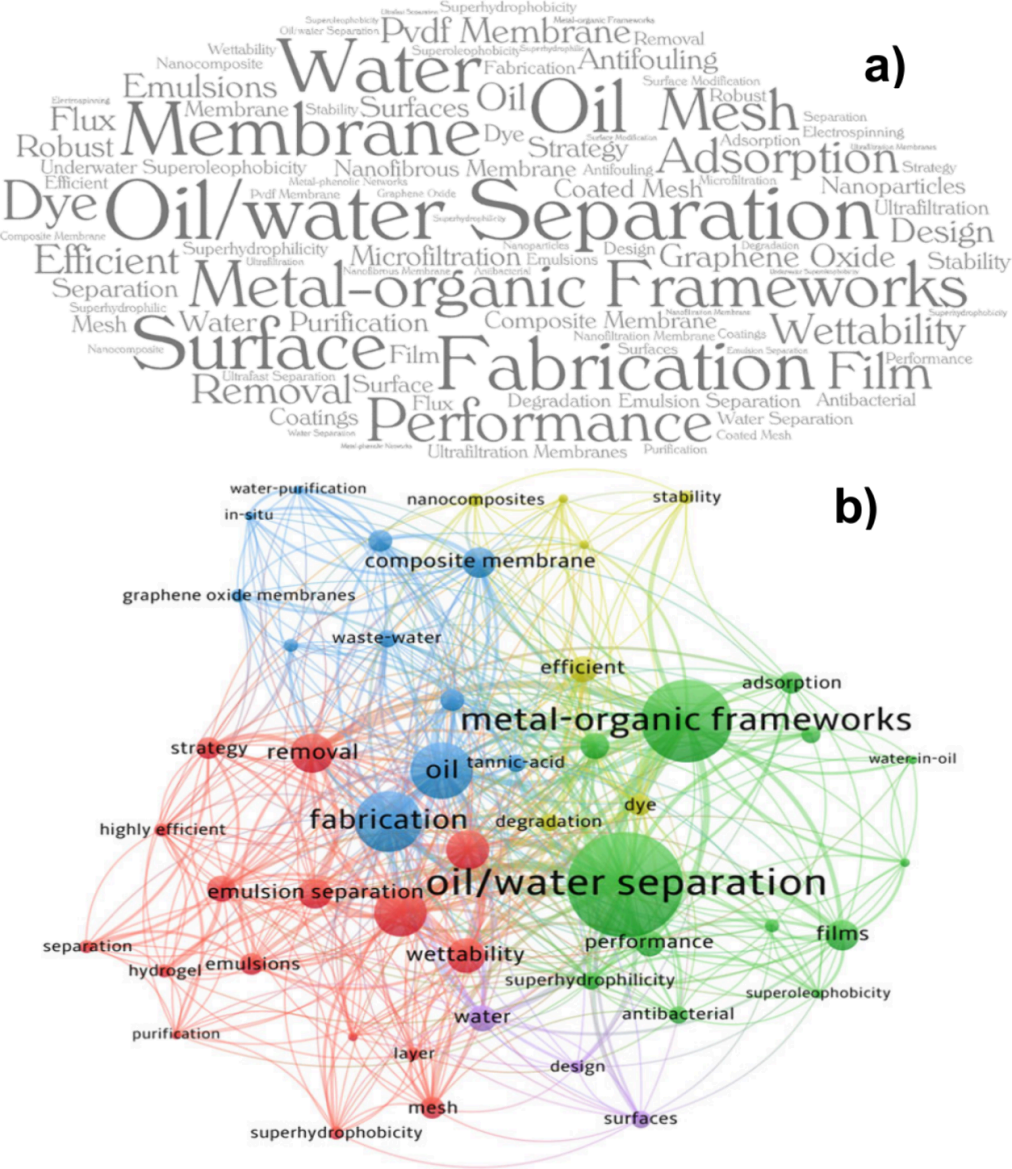
a) Display of the most used keywords in word cloud (top 50), b) Network visualization map of top 50 keywords in WoS from inception to 2023.

Keywords represent the focus of published studies and provide an idea of the topics covered within a given area. In addition to the authors, reviewers and editors can add keywords to publications. A total of 587 keywords were identified, of which 327 were correlated. However, it was selected to display only the 50 most frequently selected ones (the top 50). Figures 4a and 4b present a word cloud with the 50 keywords most frequently chosen by authors, editors, and reviewers for retrieved articles, and the network visualization map of the top 50 keywords, respectively.

As can be seen in Figure 4a, “*oil/water separation*” and similar (80 occurrences), “*membrane*” and similar (36 occurrences), “*oil*” (23 occurrences), “*water*” (22 occurrences), “separation” (11 occurrences), “*emulsion separation*” (10 occurrences), and “*emulsion*” (6 occurrences) were frequently used words, primarily due to the direct relationship with the theme.

“*Metal organic frameworks*” and similar (60 occurrences) are an emerging class of porous materials constructed from metal ions or clusters (also known as secondary building units) and organic binders.^90^ Several recovered documents refer to the application of MOF based on Fe, Cu, Co, Zn, and Zr, among others, for membrane preparation.^21−30^ Saini et al. (2022) reviewed hydrophobic-oleophilic, hydrophilic-oleophobic MOF underwater and switchable wettability MOF and their implementation as o/w separation materials.^91^Table 5 presents some MOF used, their specific properties, and the performance of MOF-based membranes in o/w emulsion separation.

**Table 5:** MOF-Based Membranes Used to Separate o/w Emulsions^21,22,24−28,76,92−102^

				Performance		
MOF	Description	Specific properties	Membrane	Separation efficiency (%)	Membrane separation flux (L/m^2^ h)	Reference
UiO-66	Typical Zr-based MOF, commonly synthesized using 1,4-benzenedicarboxylic acid as ligand.	Hydrophilicity, high stability in water, high water adsorption capacity, high roughness, functionalizable.	Poly(acrylic acid) (PAA)	99.9	2,330	(21)
			Polyvinylidene fluoride (PVDF) modified with PVA	99	15,600	(28)
HKUST-1	Cu-based MOF with organic ligands of benzene-1,3,5-tricarboxylic acid, forming a highly porous three-dimensional crystalline structure.	Hydrophilicity, high stability in water, photocatalytic activity.	Stainless steel (SS) mesh	99.99	300	(95)
MIL-53 (Fe)	Fe-based three-dimensional MOF with 1,4-benzenedicarboxylate ligand.	Hydrophilicity, high stability in water, photocatalytic and adsorptive activities.	Polydopamine (PDA) modified with reduced GO	98.12	539.79	(24)
ZIF-8	Zn-based MOF with imidazolate ligand.	Hydrophobicity, moderate stability in water, high chemical and thermal stability, functionalizable.	Polyacrylonitrile (PAN)	99.9	>900	(76)
			PVDF	92.93	1.11	(97)
MIL-100 (Fe)	Fe-based MOF linked to 1,3,5-benzotricarboxylic acid.	Hydrophilicity, good stability in water at neutral pH, catalytic and photocatalytic ability, and high adsorption capacity.	Graphene oxide (GO)	>99	12,457	(27)
ZIF-67	Co-based MOF linked to dimethylimidazole.	Hydrophobicity, catalytic capacity, excellent chemical and thermal stability, and functionalizable.	Two-dimensional transition metal carbides (MXene) modified with sepiolite	99.4	787.3	(100)
MIL-88B	Fe-based MOF with terephthalic acid hydrate ligands.	Hydrophilicity, and photocatalytic activity.	GO with g-C_3_N_4_	99.21	1,611 ± 63	(101)
MIL-88A	Fe-based MOF with terephthalic acid ligand.	Hydrophilicity, photocatalytic and adsorptive activities, and antifouling performance.	Stabilized PAN with GO	>99.2	920–7,083 ± 47	(102)

“*Wettability*” (17 occurrences), “*antifouling*” and similar (13 occurrences), “*underwater superoleophobicity*” (12 occurrences), “*superhydrophilicity*” (8 occurrences), “*superhydrophobicity*” (8 occurrences), “*superoleophobicity*” (7 occurrences) and “*antibacterial*” (6 occurrences) are some prevalent properties desired for membranes used in the separation of emulsions, and for this reason, several of the documents presented these keywords.^21,30,68−72^ The wettability, superhydrophobicity, and underwater superoleophobicity are related and depend on the physicochemical properties of the membrane, considering the contact angles with water.^12^

It is crucial to highlight the role of nanotechnology in this field, as evidenced by the frequent occurrence of terms such as “*nanofibrous membrane*” and related terms (14 occurrences), “*nanoparticles*” (9 occurrences), and “*nanocomposite*” (7 occurrences). This trend reflects the substantial investments in nanotechnology by leading countries, particularly China and the USA, which are at the forefront of publications in this area.^52^

Additionally, keywords related to membrane preparation materials have been widely observed, including “*PVDF membrane*” and related terms (15 occurrences), “*mesh*” and similar terms (14 occurrences), “composite membrane” and related terms (14 occurrences), “*graphene oxide*” (13 occurrences), “*ultrafiltration membranes*” and similar terms (10 occurrences), “*microfiltration*” and similar terms (10 occurrences), and “*ultrafiltration*” (7 occurrences).

The keywords were divided into five clusters separated by the colors red, green, blue, yellow, and purple (Figure 4b). Keywords in the same cluster typically have closer links. Larger nodes indicate more words and grow proportionally with the citations. An increased line weight indicated a more significant co-occurrence between the two keywords. The distance between keywords reflects their relationships. The red cluster contains a significant number of keywords.^16^ The green cluster has 13 items, among which “*oil/water separation*” and “*metal–organic frameworks*” received more attention. The blue, yellow, and purple clusters contained 11, 7, and 3 items, respectively.

The network visualization map (Figure 4b) shows that the words that were already cited had high total link strength. As expected, the “*oil/water separation*” had the most significant total link strength.^101^ Keywords like “*metal–organic frameworks*″,^89^ “oil”,^79^ “*fabrication*″,^68^ and “*removal*”^58^ are next on the list.

## 3 Main Metals Used to Modify Membranes

**Figure 5:**
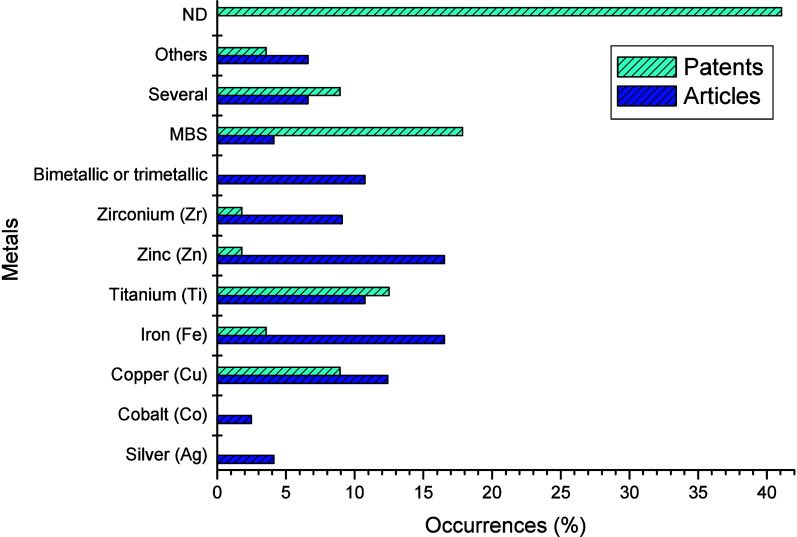
Main metals contained in the membranes in documents recovered from WoS and DII databases. ND: Metal not defined; Others: Metals other than the related categories; Several: Application of more than one metal to individually modify each reported membrane; MBS: Metal was the substrate.

The use of metals is a widely used strategy for changing the membrane surface, which may impart hydrophilic properties and reduce the problems of organic deposits.^103−105^ Metallic materials with special wettability for separating o/w emulsions have been extensively studied as substrates for filter membranes.^38^ Articles and patent filings retrieved from the WoS and DII searches were categorized according to the metals contained in the membranes. The results are shown in Figure 5.

As shown in Figure 5, Fe, Zn, and Cu were the most used metals for modifying the membranes, with occurrences of 16.53%, 14.53%, and 12.40%, respectively. Owing to the secrecy of the patents, 41.07% did not have the metal defined, and 17.86% did not change it, but it was the substrate (MBS). Ti stood out and was present in 12.50% of the documents. Several articles presenting membranes containing two or three metals (10.74%) were also recovered.

Owing to the catalytic or photocatalytic properties of Fe, Cu, and Ti, modification with these metals endowed some of the prepared membranes with an extra capacity to carry out the degradation/oxidation of organics,^22,30,77,103,106−109^ as well as self-cleaning, favored by the generation of reactive radicals in the reactions involved. Some formed membranes, such as those modified with Ag, may be antibacterial.^32,73,110^

He et al. (2022) reported the synthesis of a Cu-based MOF with two-dimensional hierarchical structures grown directly on a copper mesh.^23^ The material exhibited underwater superhydrophilicity and superoleophobicity, allowing o/w separation by gravity and achieving an ultrahigh flow. The Chinese patent CN112973470-A reports the synthesis of a membrane of a metal mesh base material modified with a functional fabric comprising non-cross-linked material dispersed in cross-linked material with high water permeability and penetration pressure.^111^ Excellent corrosion and wear resistance are some of the advantages of these materials.

A hydrophilic, visible-light-responsive nanofiber membrane was fabricated by introducing an Fe-containing MOF into the three-dimensional matrix of a hydrolyzed PAN membrane. Among the enhanced properties, it is worth mentioning the excellent separation efficiency, high permeation flux, and photo-Fenton activity.^107^ Similarly, Guha et al. (2017) modified the surface of commercial polysulfone (PSf) membranes with PDA and copper oxide (CuO) and manganese dioxide (MnO_2_) nanoparticles, thereby providing a membrane with the ability to catalyze the degradation of hydrogen peroxide (H_2_O_2_) and minimize the deposition of organics.^103^

Li et al. (2021) fabricated a mesh decorated with an Ag and CuO heterostructure endowed with super hydrophilicity, underwater superoleophobicity, and photocatalytic performance.^106^ Yin and Meng (2022) prepared a chitosan/aminographene/titanium dioxide (TiO_2_) self-cleaning photocatalytic superhydrophobic mesh membrane with excellent continuous o/w separation.^112^ Ding et al. (2020) manufactured a nanofibrous membrane subsea superhydrophilic and superoleophobic with emulsion separation efficiency greater than 99% and excellent antibacterial properties against *E. coli*.^32^

The use of Zn-, Zr-, and Co-based MOF is significant among recovered documents. Mai et al. (2020) prepared a graphene membrane modified with PDA and Zn-based MOF, which showed wettability and allowed the separation of liquid mixtures of different ranges.^113^ Samari et al. (2020) synthesized a poly(ether sulfone) (PES) UF membrane with thermal stability and high permeability using melamine-modified Zr-based MOF, which showed improved antifouling properties.^25^ Liu and Cao (2021) designed a mechanically durable nylon mesh with carboxymethyl chitosan to integrate with a Co-based MOF with great application potential for filtration and separation in complex environments.^114^

Despite the great attention given to the application of MOF in the removal of pollutants in the liquid phase, Li et al. (2020) reported challenges such as improving the capacity of existing MOF, low stability when using such materials in a real environment, and high cost, suggesting that the improvement of simpler synthetic methods and easier regeneration can help address these issues.^37^

Some authors have developed methods by varying the metals incorporated into the membranes. Ma et al. (2020), for example, described the preparation of nanofibrous membranes multifunctional superhydrophobic introducing plant polyphenol metal complexes with Al, Ag, Cu, and Ti into electrospun polyimide (PI) modified with poly(dimethylsiloxane) (PDMS).^73^ In addition to its excellent separation performance, the Al-containing membrane exhibited anti-impact, low adhesion, self-cleaning, antibacterial activity, good biocompatibility, robust mechanical strength, exceptional UV protection, and resistance to various adverse conditions.

Shen et al. (2021) reported the synthesis of monophenol-based materials with Fe, Cu, and Ni, which endowed substrates such as polypropylene (PP) microfiltration (MF) membranes with underwater superoleophobicity.^115^ Chromium (Cr), magnesium (Mg), nickel (Ni), tungsten (W), Al, and Mn were less common in recovered articles and patents.

In reported systems, metals and MNP have a positive impact on the separation of o/w emulsions, acting mainly as fouling mitigating agents.^116^ The main mechanisms involved in the separation of o/w emulsions are size exclusion and adsorption.^104^ However, strategies such as the addition of light, combined or not with oxidants such as H_2_O_2_, make the system capable of oxidizing pollutants.^104−109,109^

Metals and MNP help to modify the surface affinity of the material, adjust the size and distribution of pores, and promote greater ease in the destabilization of emulsions by affinity with specific pollutants. Chemical and electrostatic interactions can occur because of the charge on the membrane surface. Surface modification promotes antifouling properties and changes in the surface roughness, further influencing the interaction between oil and water.

Membranes conventionally perform selective separation under the action of a driving force. In filtration with porous membranes using pressure, the liquid passes from one side to the other, with the larger solutes being retained, whereas the solvent permeates along with the small molecular solutes, enabling separation and concentration.^104,117^

Larger pollutants are retained on the surface, while smaller ones flow through irregular pores, where they collide with the walls and can be adsorbed on the attached metals, aided by interaction forces, such as van der Waals forces.^104,118^ Selective adsorption facilitates the removal of smaller compounds or stable emulsions not easily separated by size exclusion.

Organic compounds on the membrane surface can be oxidized to achieve a self-cleaning effect.^109^ In the case of membranes based on the Fenton process, for example, hydroxyl radicals (•OH) are generated from the decomposition of H_2_O_2_ added to the system, catalyzed by iron(II), leading to the mineralization of oily pollutants, according to the eqs 1-11.^119−121^ It is worth noting that a Fenton-like reaction can occur for the generation of reactive radicals using materials containing Cu, Co, or even Ni via similar mechanisms.^122−124^
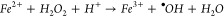
1
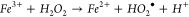
2
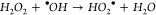
3
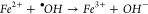
4
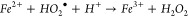
5
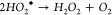
6
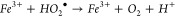
7
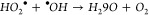
8
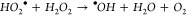
9
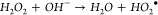
10
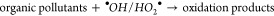
11

When membranes containing photocatalytic materials (PM), such as TiO_2_, are exposed to light, photoelectrons and holes (e^–^/h^+^) are generated. Both can be transferred to the membrane surface, where they react with molecules present, such as water and oxygen, generating ^•^OH and superoxide radicals (O_2_^•–^), mainly, which attack and degrade pollutants present in the fluid in contact with the membrane. This mechanism can be observed in eqs 12–21.^125^ UV light endows membranes with superhydrophilicity by decreasing the water contact angle, enabling water permeation and pollutant rejection.^126^
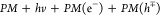
12
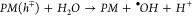
13
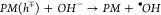
14
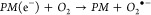
15
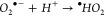
16
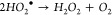
17
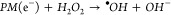
18
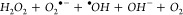
19
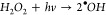
20
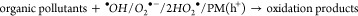
21

The use of other oxidants such as persulfates and ozone (O_3_) has been reported.^127−130^ Persulfate activation generates additional sulfate radicals (SO_4_^•–^) with high oxidation potential (2.5–3.1 V).^120^ However, SO_4_^•–^ have the disadvantage of requiring post-treatment to meet the discharge demand.^131^ In the case of ozonation, oxidation is rapid under mild conditions, but the O_3_ utilization rate is low.^132^ More efforts are needed to accelerate the application of such technologies to the treatment of complex effluents, such as PW.

For membranes where the metal is the substrate, compared to membranes where the metal or MNP is incorporated/deposited, the metal plays a passive role. Size exclusion occurs in porous membranes; however, the presence of an active layer determines whether new functionalities are added, such as catalysis or changes in chemical affinity.^104−109^

As previously reported, the modification of membranes with metals or MNP, in general, presents advantages, such as increased separation efficiency, in some cases, catalytic or photocatalytic activity, and antifouling properties, in addition to frequently presenting antimicrobial properties and improved permeability. However, it is worth mentioning that the cost associated with the modification, the leaching of metals into the permeate, the complexity of the processes for obtaining the materials, and the toxicity of the metals used to modify membranes can be considered limitations to the use of such a strategy, motivating the study of more efficient approaches.

## 4 Main Base Substrates and Membrane Preparation Approaches

**Figure 6:**
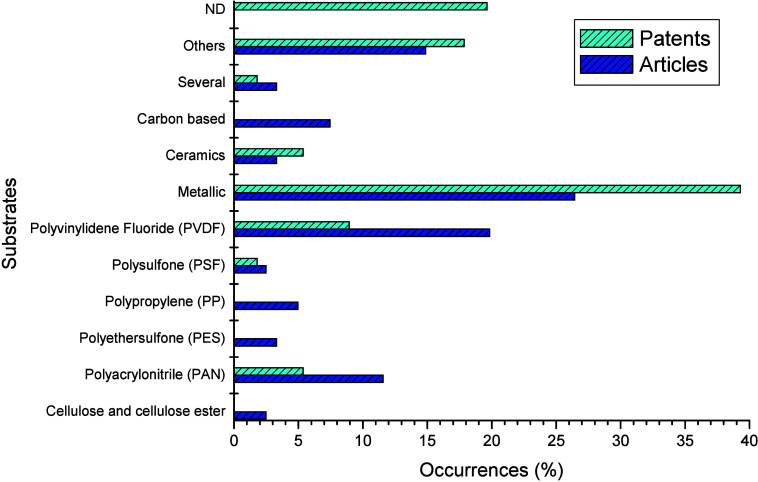
Main substrates used to prepare membranes in documents recovered from WoS and DII databases. ND: Substrate not defined, Others: Substrates other than the related categories, Several: Application of more than one metal-modified substrate reported in the same document.

The choice of substrate and the approach to membrane preparation are crucial for the morphology and properties of the obtained materials. The articles and patent filings retrieved from WoS and DII searches were categorized in terms of the substrates used to prepare the membranes. The results are shown in Figure 6.

As shown in Figure 6, organic polymers are commonly used to prepare membranes. This is mainly because of their greater flexibility, good film-forming properties, high selectivity, and inexpensive materials for preparation.^16^ As a trend in the articles, great emphasis can be given to PVDF (19.83%), PAN (11.57%), and PP (4.96%). Metals (26.45%) and carbon-based materials (7.44%) were also selected. A similar trend was found in patents, with emphasis on polymers PVDF (8.93%), PAN (5.36%), metals (39.29%), and, due to the secrecy of the patents, unspecified substrates (19.64%).

PVDF, PAN, and PP polymeric membranes have excellent thermal, mechanical, and chemical resistances, which justifies their use.^16,104,133,134^ One of the main problems is membrane fouling, which can occur because of the hydrophobic nature of the membrane, which facilitates the deposition of organics. This problem can be overcome by modifying the surface with hydrophilic particles or nanoparticles.^104,105,135^

The choice of metals as substrates is based on their high associated efficiency, portability, high plasticity, high thermal stability, and low cost.^38^ Carbon-based materials, such as graphene oxide (GO), nanotubes, fibers, and carbon nitride, are known for their low production cost, high purity, easy scalability, and excellent chemical stability.^16^

The Chinese patent CN112871146-A presents a PVDF membrane modified by a hydrophilic adhesive and a metallic structure to facilitate the adsorption of organic pollutants, in addition to oil–water emulsion separation.^136^ Gao et al. (2021) developed a PVDF membrane containing Cu complexed with tannic acid (TA), which improved the surface roughness and membrane wettability.^137^

Zhang et al. (2022) designed a hydrophilic Fe-based MOF catalyst with GO to impregnate a stabilized PAN nanofibrous membrane, which exhibited underwater superoleophobicity, robust mechanical strength, self-cleaning, and photo-Fenton activity.^109^ The Chinese patent CN112726028-A deals with a superhydrophilic composite nanofiber membrane with a rough structure, containing a membrane material formed by weaving and cross-linking PAN composite fibers modified by PVA and alkali metals.^138^

Yu et al. (2019) modified a PP membrane with juglone and Fe, which exhibited underwater superhydrophilicity and superoleophobicity, a high separation efficiency, and good reuse for various o/w emulsions.^139^ Cai et al. (2019) prepared a PDA-modified PP membrane and Zn-MOF to obtain a material with superhydrophobicity and a durable coating owing to its repairability.^140^

Metallic materials have been used as substrates in the oldest patent. This patent reports the use of superhydrophobic/superoleophilic SS or metallic copper fibers coated with perfluoroalkyl copolymer siloxane.^42^ The Chinese patent CN114288713-A reports a stainless-steel mesh with an Al-based MOF layer with surface wettability.^141^ Switchable superwetting of Zn-based MOF-modified copper core/shell nanowire membranes was reported by Li et al. (2018).^142^

Among the GO membranes, it is possible to highlight a stable multifunctional membrane of reduced GO modified with PDA and an Fe–Co-based MOF.^42^ Wang et al. (2022) report the synthesis of a GO nanosheet containing a Fe-based MOF with super hydrophilicity and demulsification functions endowed with the antifouling capacity and ultrahigh flow for emulsion separation.^27^

Despite their limited permeability and high price, ceramic membranes exhibit high selectivity and structural, mechanical, and thermal resistance.^16^ Jiang et al. (2022) presented the synthesis of a nanoporous membrane derived from solid mining waste, which confirmed such properties.^46^ A porous membrane of silicon carbide (SiC) bonded with mullite, prepared using coal ash as an additive, was reported by Das et al. (2021) and is applicable for oily effluent treatment with sustainable bias.^143^

The choice of the membrane preparation method is essential, primarily because it influences the morphology and separation performance. The approaches applied to membrane preparation are mixing (M), which uses methods such as phase inversion, electrostatic spinning, and sol–gel processes; surface coating (SC), with techniques such as spin coating, immersion precipitation, and vacuum filtration; and bottom-up synthesis (BS), with layer-by-layer assembly, chemical grafting, and chemical vapor deposition methods.^104^ The phase inversion method is the most common and relevant method.^16^

In general, the M approach is the simplest and most cost-effective approach, allowing direct incorporation; however, it suffers from challenges regarding uniform dispersion and control of specific properties.^42,48,146^ The SC approach, despite offering greater control over surface functionality, depends on good adhesion conditions.^68,144,145^ BS, however, provides more precise control, allowing the creation of membranes with optimized structures and functionalities at the molecular level, but the process is slow, complex, and costly.^47,141,147^Table 6 presents advantages and disadvantages, and information on cost, scalability, reproducibility, and impact on membrane properties.

**Table 6:** Comparison between Membrane Preparation Techniques^42,47,48,68,104,141,144−147^

Technique	Advantages	Disadvantages	Cost	Scalability	Reproductivity	Impact on membrane properties
Phase inversion	Well-established.	Slow membrane formation.	Moderate to high	High	Good	High selectivity and adjustable permeability, improved chemical resistance with well-dispersed MNP.
	Simplicity and ease of operation.	Use of excess chemicals.				
	Applicable on an industrial scale.	Lower production capacity of highly porous surfaces compared to electrostatic spinning.				
Electrostatic spinning	Simple device.	Slow production.	Moderate to high	Low	Average	Nanofibers with high surface area and permeability. Improved antimicrobial and catalytic properties.
	Controllable process.	Low product yield.				
	Production of nanofibrous membranes with a high surface area.	Sensitive to nanoparticle dispersion.				
Sol–gel processes	Homogeneity in the incorporation of metals/MNP.	Complex process and sensitive to synthesis conditions.	High	Low	High	High selectivity and thermal or chemical stability due to the homogeneous incorporation of metals/MNP.
	Metal/MNP loading adjustment.	Difficulty in obtaining large areas of thick membranes.				
	Production of membranes with high thermal and chemical resistance.	Time-consuming for solvent evaporation at low temperatures.				
Spin coating	Precise control of layer thickness.	Difficulty in creating thick structures.	Moderate to high	Low to moderate	High	Produces highly uniform layers with optimized functionalities such as catalytic and antimicrobial properties.
	Production of layers with excellent uniformity.	Requires specialized equipment.				
	Ideal for creating high-density membranes.	Not applicable to membranes with complex geometries.				
Immersion precipitation	Widely used and well-established process.	Less control over particle uniformity and distribution.	Moderate	High	Average	Fabrication of symmetrical or asymmetrical membranes with adjustable properties.
	Easy control of preparation conditions.	Unstable membranes.				
	Possibility of selectivity and permeability control.	Use of excess chemicals.				
Vacuum filtration	Simplicity and efficiency of thin membrane preparation.	Difficulty in obtaining uniform membranes.	Low	Low	Average to good	Improvements in structural uniformity, mechanical properties, functional efficiency, and chemical stability.
	Suitable for the direct incorporation of particles into the matrix.	High energy consumption.				
	Does not require the use of complex solvents.	Low productivity on a large scale.				
Chemical graft	Allows highly specific and selective functionalization.	This process involves several steps.	Moderate to high	Low	Average	Introduces specific properties, such as chemical or catalytic affinity for metals/MNP.
	Suitable for introducing functional groups for particle anchoring.	Requires specialized reagents and multiple steps for optimization.				
	High stability of produced membrane.	High consumption of metals/MNP.				
Chemical vapor deposition	High precision in metal/MNP deposition.	Use of advanced equipment and high-purity materials.	Very high	Low	High	Allows the creation of membranes with ultrathin coatings, high selectivity, and thermal/chemical resistance.
	Production of extremely chemically and thermally resistant membranes.	Limitations of the use of polymeric substrates that are sensitive to the conditions employed.				
	Excellent for creating thin, homogeneous metallic coatings.	High energy costs and dependence on reactive gases.				
Layer-by-layer assembly	Versatility allows for thickness and composition adjustments with high precision.	Relatively slow process owing to multiple deposition cycles.	Moderate to high	Moderate	High	Excellent for creating highly customized layers with tunable properties such as selective adsorption and surface functionalization.
	Cheap and flexible.	Sensitive to variations in assembly conditions.				
	Suitable for controlled and uniform incorporation.	Challenging control of thick-layer formation.				

The costs associated with obtaining membranes using techniques such as phase inversion, immersion precipitation, and layer-by-layer assembly are mainly justified by the use of polymers and solvents. Electrostatic filtration, sol–gel process, spin coating, and chemical vapor deposition use specialized equipment or materials. In addition, the layer-by-layer assembly and chemical grafting depend on the number of steps. Scalability and reproducibility depend on the complexity and scale of production, as well as the control of the production of materials.

Using simple vacuum filtration, a well-known SC approach, Zhu et al. (2022) prepared a PDA-modified mixed cellulose ester membrane containing sepiolite and Ti.^68^ Yang et al. (2022) fabricated a nanostructured porous membrane material by depositing a GO complex onto metal strands that exhibited corrosion resistance.^144^ Using the immersion method, Bao et al. (2022) prepared a PSf UF membrane coated with a phytic acid-Fe complex, defined by the authors as a quick, easy-to-scale, and economical method.^145^

Using the electrospinning method, Wu et al. (2022) obtained a poly(ether imide) (PEI) nanofiber membrane with Cu-based MOF nanoparticles, which showed selective wettability, structural stability, and high separation efficiency in complex environments.^48^ Zhang et al. (2022) prepared Cu-containing PAN UF membranes using the phase inversion method.^146^ The Chinese patent CN1721030-A reported membrane preparation based on steel or copper mesh coated with perfluoroalkyl copolymer siloxane using a sol–gel process.^42^

Su et al. (2022) described the layer-by-layer assembly of PVDF nanofiber membranes electrospun with Ag-thiol nanoparticles.^47^ Gao et al. (2020) synthesized a PP membrane with a Zr-based MOF and confirmed the chemical bonds between the metal and polymeric substrate.^147^ The Chinese patent CN114288713-A used the same approach to synthesize a stainless-steel mesh membrane with an Al-based MOF.^141^

BS is the most recent approach for membrane preparation. The most relevant feature is obtaining membranes that are more stable than those obtained using other approaches.^104^ This is justified by the fact that bonds are formed between the active components of the membranes and the metal, making it resistant to leaching into the medium.

Substrates such as melamine sponge, mesh and cotton fiber, carbonized wood, nylon, poly(arylene sulfide sulfone), polyacrylic, poly(lactic acid), poly(l-lactic acid), polystyrene, polyurethane, PDA, PDMS, PEI, PI, PVA, Bombyx silk Mori, linen fabric, polypyrrole, and chitosan were cited less frequently in articles and patents.

## 5 The Role of Membranes Containing Metals or Metal Nanoparticles in o/w Emulsion Separation for Sustainable Development

Recently, the United Nations adopted a global action plan titled “Transforming Our World: The 2030 Agenda for Sustainable Development”. It comprises 17 Sustainable Development Goals (SDG) and 169 interconnected targets, serving as a call to action for all countries, recognizing that the challenges faced by the world require an integrated approach.^148^

Oily wastewater is mutagenic and carcinogenic to humans, inhibiting plant growth and increasing BOD and COD. It also provides an impermeable layer for sunlight, preventing light penetration into aquatic ecosystems, which affects aquatic life when discharged without treatment.^9,149^ These characteristics render it a dangerous contaminant for drinking water, underground water resources, air, and agricultural production. Disposal of untreated oily wastewater into different environmental compartments can negatively impact the achievement of SDG 3 (good health and well-being), SDG 14 (life below water), and SDG 15 (life on land).

Advancing technologies that improve o/w emulsion separation and reduce O&G concentrations can help achieve the SDG goals. Regarding SDG 3, reducing O&G ensures better health outcomes for the population, especially because it does not compromise the supply of clean drinking water. SDG 14 and 15 can also be supported, as properly treated oily effluent discharge does not harm aquatic life or affect soil when reused for irrigation. Furthermore, SDG 9 (industry, innovation, and infrastructure) and 11 (sustainable cities and communities) can benefit from the ongoing development of more sustainable and innovative technologies.

Although the promotion of several SGD is possible, environmental impacts may occur throughout the life cycle of the membrane. Among them, the release of metals or MNP into the permeate stream stands out as they can be toxic to aquatic organisms or accumulate in soils and sediments when discarded, generating long-term impacts. Byproducts can be generated during the operation of catalytic membranes, and worn metals or MNP can accumulate in solid waste, making it difficult to properly manage these materials.

Regarding economic impacts, the use of noble metals or even advanced nanoparticles, as well as complex manufacturing processes, can increase production costs. The loss of efficiency requires frequent replacements, which makes the application of this technology expensive. Additionally, the costs associated with safe disposal, especially in the face of increasingly stringent regulations, represent a point of concern.

Various membranes containing metals or MNP for o/w emulsion separation have demonstrated high efficiencies. The reuse of treated water aligns with sustainability principles and contributes to water conservation, reduced environmental pollution, the preservation of natural resources, cost savings, and improved energy efficiency. However, it is estimated that less than 1% of the total volume of oily water is reused in the upstream and downstream oil and gas exploration processes.^150^ Thus, developing more efficient membranes could play a crucial role in responsible management practices by providing both environmental and economic benefits. Improved life cycle analysis of membranes containing metals or MNP can be a valuable tool for identifying and mitigating impacts along the entire value chain.

## 6 Current and Future Developments

The analysis of the current and future development of membrane technology containing metals or MNP for the separation of o/w emulsions, especially in the context of PW treatment, reveals a promising scenario, but is full of challenges.

Membrane processes have emerged as viable alternatives for the separation of o/w emulsions, mainly using modified membranes. These membranes, which incorporate metals or MNP, exhibit improved properties, including resistance to fouling, by both organic materials and biological deposits.^13,17^ However, there is still a significant need for research to better understand the mechanisms that contribute to these improvements and how to maximize the process efficiency.

One of the most notable advances is the antibacterial activity observed in some modified membranes, which may indicate that, in addition to separation, these membranes can act in the control of unwanted microorganisms, promoting more efficient treatment.^32,73,110^ However, understanding the mechanisms underlying this activity requires further investigation.

In addition, some studies have explored the synergy between oil separation and organic compound degradation using catalysis and photocatalysis processes.^22,30,77,103,106−109^ The possibility of a system that not only separates pollutants but also degrades them represents a significant advancement, contributing to more sustainable and economically viable solutions. This approach can reduce fouling and extend the life of membranes by taking advantage of the self-cleaning properties resulting from the generation of reactive radicals.^77^ Despite this, obtaining membranes with catalytic stability, low mechanical fragility, and chemical resistance, and the use of several operational cycles is relevant.

Pertinent research questions also include improving methods for chemically modifying metals or MNP to obtain superhydrophilic or superoleophobic surfaces, and ensuring greater selectivity and separation efficiency.^151^ Furthermore, exploring the use of MNP synthesized via eco-friendly routes, such as plant- or microorganism-mediated biosynthesis, and promoting the use of abundant or recyclable metals to reduce the environmental impact and associated costs is timely.^152,153^ The development of modular systems that integrate these membranes into industrial oily water treatment processes requires further development.^154^

A constant challenge in membrane development is metal leaching, which compromises the process efficiency and can cause pollution. Techniques such as BS have shown the potential to create more durable materials and minimize leaching to mitigate this problem.^104^ Homogeneity in the distribution of metals on the membrane surfaces is also crucial. Therefore, exploring new manufacturing techniques and combining different methods is an effective strategy.

More specific challenges include the handling of complex emulsions, such as those stabilized by surfactants or those with very small droplet sizes, motivating the use of innovative strategies.^155^ The cost of specific MNP, such as Ag, limits large-scale applications, motivating studies that avoid material loss during operation.^156^

Currently, China stands out as a leader in the production of articles and patents in this area, which reflects not only significant investment in research but also the creation of nanotechnology centers at universities.^50−52^ This infrastructure is expected to drive innovation and increase the number of patents in the future. However, most studies still focus on laboratory scales, which limits the transfer of these innovations to large-scale applications.

Therefore, the future of membrane technology containing metals or MNP for the treatment of oily effluents from the oil industry is promising but requires continued effort to overcome technical challenges and expand applied research. The combination of advances in nanotechnology and manufacturing processes and a deeper understanding of the mechanisms involved will be key to the evolution of this technology and its effective implementation in water treatment.

## 7 Conclusion

In the present study, it was found that membranes containing metals or MNP for the treatment of oily effluents constitute an emerging area of research in the scientific community.

China and, very timidly, the USA were the primary producers of the recovered documents. The temporal evolution of article publication and patenting analysis revealed more significant growth in interest in the area from 2017 to 2018, with the number of patent filings being lower than the number of article publications. The main areas involved in the articles were Engineering, Materials Science, and Chemistry, whereas for patents, the main areas were Chemistry, Polymer Science, and Water Resources.

Chinese institutions are among the primary producers of the recovered documents. In the case of patent filings, the leading company is the China Petroleum & Chemical Corporation (SINOPEC), which, in partnership with the Sinopec Beijing Research Institute of Chemical Industry, has granted four deposits.

Analysis of the recovered documents indicated the significant use of Fe, Zn, Cu, and Ti to modify membranes, metallic materials, PVDF, PAN, PP polymeric membranes, and carbon-based primary substrates. Documents regarding ceramic materials have also been reported and evaluated.

Finally, it is possible to conclude that this is a developing area given its recent development. The involvement of nanotechnology, already presented in several publications, is expected to become increasingly significant, further improving the characteristics of these materials.

## 8 Methodology

A bibliometric analysis was performed to obtain an overview of the research conducted in the scientific community. Data were collected from the WoS database. This base includes research in science, social sciences, arts, and humanities, providing indexed articles and cited references from all periodicals that are part of it.^157^ Only scientific articles published in English until July 2023 were included in the present study. The keywords were searched in the title and abstracts of publications, being “oily effluent” (or synonyms such as “refinery effluent”, “oilfield water”, “oily produced water”, “oil-field water”, “oily wastewater”, “oil-water”, “oily wastewater” and “produced water”), “membrane” and “metal”. So that the documents could be analyzed as to their suitability to the theme, these were exported to an Excel worksheet with all the fields provided by WoS. The verification consisted of reading the abstracts and sometimes the content to obtain information on the metals, substrates, and the membrane preparation approach. The resulting articles were selected for statistical analysis of the temporal evolution of publications, geographic distribution, affiliations, thematic areas, most-cited articles, and keywords. A map was constructed using the geographic distribution of the publications (Figure 2a) in Excel. The construction and visualization of the bibliometric maps (Figure 2b and 4b) were performed using VOSviewer 1.6.18. The Word Art Web site was used to produce a word cloud that contained the top 50 keywords.

The DII database was used to evaluate patent applications during the same period. This base has a roof worldwide in patent documents, presenting information from 59 issuing agencies from 1963 to the present.^158^ We used the same keywords used in the article search, combined with the Derwent Manuals D04-A* (Separation, Treatment and Purification Processes for Water Treatment), J01-D* (liquid treatment), and J01-F* (particle separation of suspended liquids), provided by the database. Thus, patent filings can be analyzed based on their suitability for the theme exported in txt files with all fields provided by the DII and imported into an Excel worksheet. This study analyzed the temporal evolution of deposits, geographic distribution and affiliations, thematic areas, and the most-cited patents.
